# Supplementary material for: Hyperspectral Ophthalmoscope Images for the Diagnosis of Diabetic Retinopathy Stage
Source: J Clin Med. 2020 May 26;9(6):1613. doi: 10.3390/jcm9061613 (PMC7356238; doi:10.3390/jcm9061613)
Supplement: Supplementary file 1 [file jcm-09-01613-s001.pdf]

Journal of
Clinical Medicine

MDPI

Article

Hyperspectral Ophthalmoscope Images for the Diagnosis of Diabetic Retinopathy Stage

Hsin Yu Yao [1], Kuang-Wen Tseng [2], Hong Thai Nguyen [3], Chie-Tong Kuo [4] and Hsiang-Chen Wang [3,*]

[1] Department of Ophthalmology, Kaohsiung Armed Forced General Hospital, Kaohsiung City 80284, Taiwan
[2] Department of Medicine, Mackay Medical College, 46, Sec. 3, Zhongzheng Rd., Sanzhi Dist., New Taipei 25245, Taiwan
[3] Department of Mechanical Engineering and Advanced Institute of Manufacturing with High tech Innovations, National Chung Cheng University, 168, University Rd., Min Hsiung, Chia Yi 62102, Taiwan
[4.] Department of Optometry and Innovation Incubation Center, Shu-Zen Junior College of Medicine and Management, Kaohsiung 821, Taiwan

S1. Fluorescein Angiography (FA)

In fluorescein angiography (FA), a water-soluble fluorescer is injected into a vein in an upper arm. The fluorescer circulates from the vein to the heart, and the heart in turn circulates the fluorescer throughout the arteries in the body. When the fluorescent dye passes through retinal blood vessels, a special camera is used to emit blue light onto the eye. The fluorescent dye absorbed in the eye then emits green light, enabling the camera to record blood vessels and blood flow in the retina on a negative film.

If the blood vessels in the retina are abnormal, the fluorescent dye leaks into the retina or stains the blood vessels. Thus, FA is used to verify whether the inner wall of the retina is normal, whether the retina and the choroid have new blood vessels, or whether the retina is ischemic. These changes can be read by an experienced ophthalmologist from a photograph.

The fluorescent agent is a safe drug, showing yellowed skin several hours after injection. In the first one or two days, urine turns orange. One of ten people experiences transient nausea and vomiting. Approximately 1% of rash reactions in the skin can improve without any intervention or by applying simple medication. The dye has less than one in 100,000 chances of causing severe allergic reactions. Patients with liver and kidney dysfunction must be given reduced volume of the dye because the fluorescent agent is metabolized by the liver and kidney [R1].

S2. Ophthalmoscope

An ophthalmoscope is an ophthalmic diagnostic optical instrument used in observing and recording the condition of the fundus. It can record and preserve fundus images in black and white or color photos, and its optical design is based on the optical principle of Gullstrand's nonreflective indirect ophthalmoscope, which can directly capture the retina. Ophthalmologists can diagnose retinal diseases by using fundus images.

The human fundus does not glow even despite the fact that various types of light enter the eyeball. Light can illuminate the fundus but is weak and is; thus, inadequate for observing the fundus and renders photography impossible. The reflected light of the cornea of a human eye is much brighter than that of the fundus and interferes with the observation of the fundus. Therefore, a fundus camera must include an illumination system that brightens the fundus with strong light and an imaging system that prevents the effects of intense reflections on the cornea. A complete fundus camera optical system consists of a camera, lighting, and observation systems. Figure S1 is a diagram of a Zeiss fundus camera [R2]. The Xenon flash light source is collected by the *L1* concentrator and passes through a series of lenses and mirrors to the eyes of a patient. According to the principle of Gullstrand, *M2* is a circular mirror and thus the input of the light source is separated from the path of observation. In the case that an input source is reflected by the retina, the source passes through

the center of the mirror and displays a fundus image on the camera's backsheet. The mirrors *M3, M4*, and *L7* and the independent light source *S1* form an observation system that can observe and monitor a fundus image in real time.

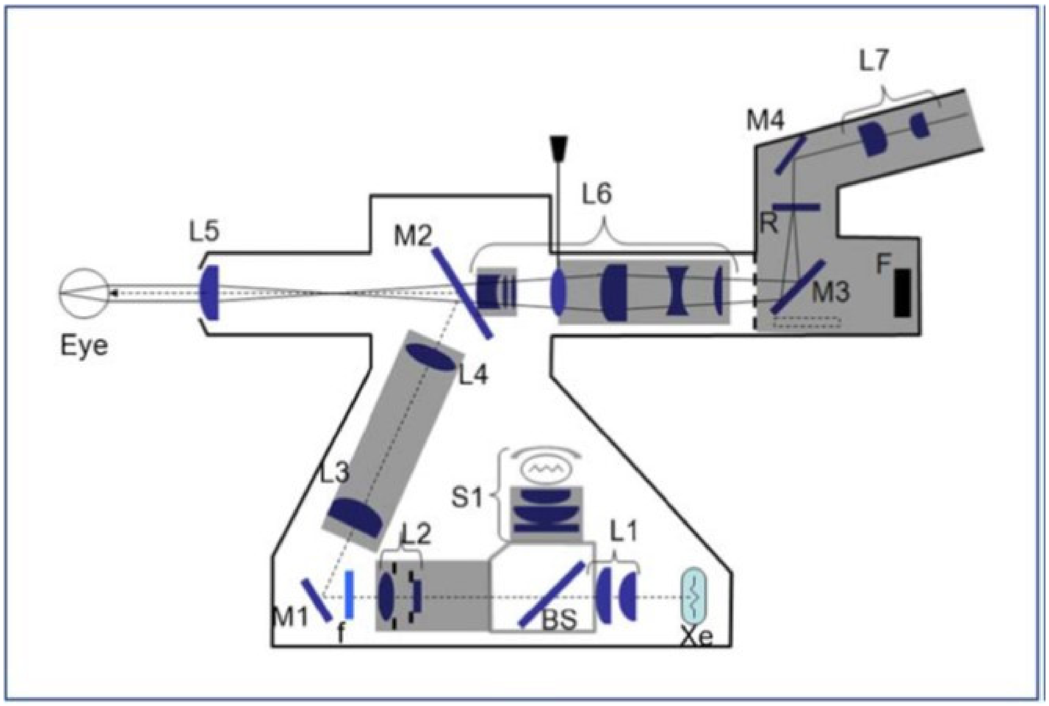

Figure S1. An image of the Zeiss fundus camera. *Xe* is xenon flash, *L1* is concentrator, *S1* is observation system light source, *L2-7* is photographic system optics, *BS* is beam splitter, *f* is filter, *M1-4* is mirror, *F* is camera negative, *R* is targeting, and the dotted and solid lines are the light source input and observation paths, respectively [R2].

S3. Optical Coherence Tomography

Optical coherence tomography (OCT) is an optical imaging technology with excellent spatial resolution for clear tissue images and has been developed for noninvasive cross-sectional imaging in biological systems. It has low coherence interference and was first proposed by Huang et al. in 1991 [R3]. At present, OCT has been widely used in clinical medicine and various fields and is mainly used to perform profile fault and spectral imaging, which is detected and processed by the scattered light spectrum and interference OCT signal [R4].

In 1993, OCT produced the earliest tomographic image in the human optic disc and the macula [R5]. OCT was originally applied to eye imaging and has a large impact on ophthalmology. OCT enables ophthalmologists to diagnose some signs. Numerous researchers are committed to OCT research, focusing on the internal eye. In 1994, Izatt et al. proposed the OCT eye structure studies in micron resolution [R6]. Bagayev proposed the application of OCT to the laser ablation of corneal thickness [R7].

A traditional OCT architecture (Figure S2) is usually built on a Michelson interferometer. A light source is struck at the reference and sample ends through a beam splitter and then reflected back. Finally, the light source falls on the photodetector to generate interference. When light strikes a tissue it scatters or is absorbed. OCT mainly collects backscattered photons for imaging. Theoretically, water molecules have a strong absorption capacity for light from 1400 to 2600 nm, and scattering angle decreases with increasing light penetration depth. Therefore, light in this band is absorbed by water molecules in biological tissues. If an ultraviolet light band below 360 nm is selected, melanin and heme easily absorb light. Therefore, most of the current near-infrared light bands (700–1300 nm) are used as light sources.

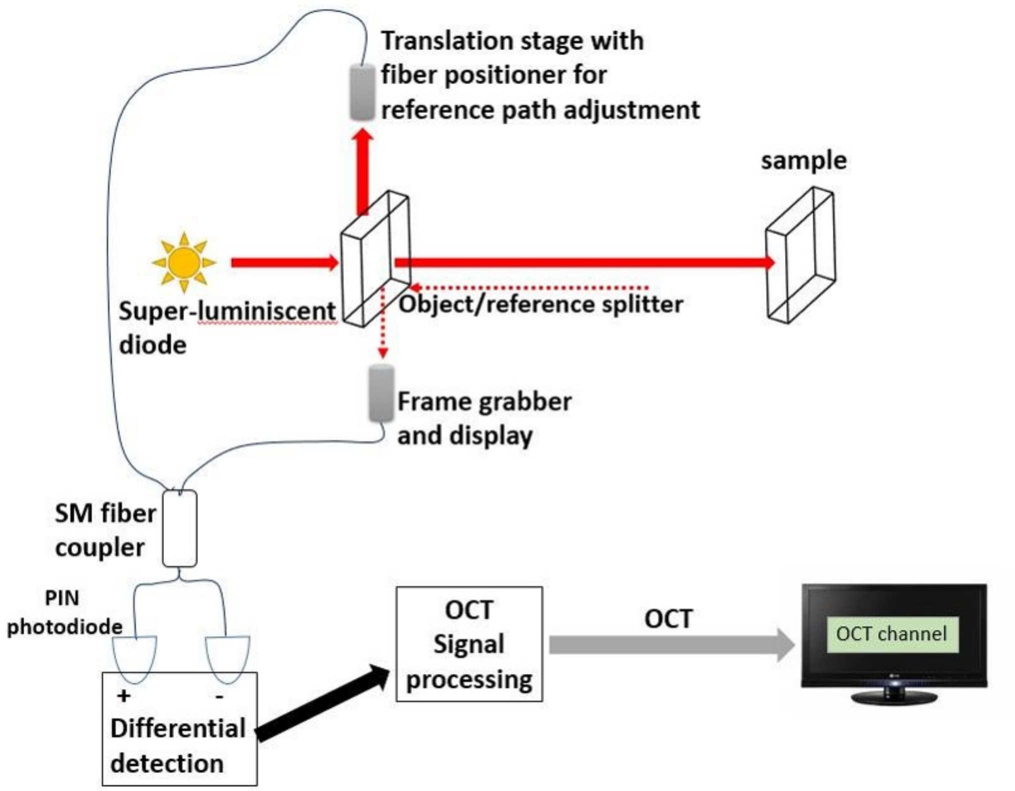

Figure S2. Traditional optical coherence tomography (OCT) architecture.

S4. Retinal Image Processing Algorithm

To automate the retrieval of vascular information in the retinal images, we used a new image processing algorithm, which is depicted in Figure 5. We divided the calculation process into the following steps:

A. Preprocessing

In a colored fundus image, the color of the blood vessel is compared with the lesion. For example, microvessels and bleeding are dark. Excluding these areas when detecting blood vessels is important because it prevents misjudgment. Only one step is used in the retinal vascular treatment in the preprocessing of an image. In the RGB images, the green channel has better contrast than the red and blue channels, as clearly shown in Figure S3. To suppress the components of the other two-color channels, we only used the green channel image in image processing.

B. Vascular Enhancement

The Gabor filter is a linear filter for edge detection. As shown in Figure S4, a two-dimensional Gabor filter has the characteristics of optimal localization in the spatial and frequency domains. This filter is similar to human biological visual characteristics and can be simply described as the spatial frequency of an image, spatial position, and direction selectivity. All Gabor filters are generated from the expansion and rotation of a mother wavelet and thus all Gabor filters are quite similar; in practical applications, Gabor filter can extract relevant features for edge detection at different scales and directions in the image frequency domain. The mathematical description of the Gabor filter is shown in Equations (9)–(10).

Real part:

$$g(x,y)=\frac{1}{2\pi\sigma_x\sigma_y}\exp\left[-\frac{1}{2}\left(\frac{x'^2}{\sigma_x^2}+\frac{y'^2}{\sigma_y^2}\right)\right]cos(2\pi f_0 x') \quad (S1)$$

Part of the imaginary part:

$$g(x,y)=\frac{1}{2\pi\sigma_x\sigma_y}\exp\left[-\frac{1}{2}\left(\frac{x'^2}{\sigma_x^2}+\frac{y'^2}{\sigma_y^2}\right)\right]sin(2\pi f_0 x') \quad (S2)$$

where $g(x,y)$ is the response value obtained by the image through the Gabor filter; σ_x and σ_y are standard deviation of the x and y directions in the Gabor filter, respectively; and f_0 is frequency of the curve. Cosine and sinusoid have a period τ (the width of the object to be extracted in the image). Moreover, $f_0 = 1/\tau$.

In this filter, 180 different 1° angles from the range of [(-π)/2, π/2] are used for edge extraction in fundus images. The coordinate transformation formula in the angle rotation process is shown from S20 to S21:

$$x' = x\cos\theta + y\sin\theta. \quad (S3)$$

$$y' = -x\sin\theta + y\cos\theta. \quad (S4)$$

where (x', y') is the coordinate corresponding to the value of θ after each rotation; 180 different angle θ Gabor filters $g(x,y)$ were used for fundus imaging to obtain a range of filter responses $G^{\theta}(x,y)$. The absolute values of the real and imaginary parts of each angle are then added, as shown in Equation (S5):

$$sqrt(G_{\gamma}^{\theta 2}+G_i^{\theta 2}) = G^{\theta}. \quad (S5)$$

where G_{γ}^{θ} is the real response at all angles, and G_i^{θ} is the imaginary response of each angle. Only the maximum response R(x, y) is preserved for the effective detection of blood vessel position at every pixel (x,y), as shown in S19:

$$R(x,y) = Max(G^{\theta}(x,y)), \theta = \left[-\frac{\pi}{2}:\frac{\pi}{180}:\frac{\pi}{2}\right] \quad (S6)$$

C. Contrast Enhancement

The brightness value of the image-specific interval is mapped to the new interval value, and the mapped curve shape is specified by the gamma parameter. In the default case, map the 1% brightness value before and after the original image to the highest and lowest intensities (discard 0%–1% and 99%–100%), and the overall intensity is adjusted proportionally (preset gamma = 1) for image enhancement.

D. Binarization

We used Otsu's binarization to determine the threshold of the image t. The maximum of the between-class variance is made because the threshold is between 1 and 254 (0 and 255 do not have to be considered). In summary, the t-value between 1 and 254 in the grayscale image must be determined. This value can be used to maximize S24-type inter-group variation.

$$P_1(t)P_2(t)\left[\mu_1(t)-\mu_2(t)\right]^2 \quad (S7)$$

where $P_1(t)$ represents the probability that the gray value of the image is less than or equal to the value of t; $P_2(t)$ represents the probability that the image gray value is greater than the t value, that is, $1-P_1(t)$; $\mu_1(t)$ represents the average value of the image gray value that is less than or equal to the value of t; and $\mu_2(t)$ represents the average value of the image grayscale value greater than the t value. Otsu's binarization divides the image points into two groups according to their gray values. The gray value of a group of images is smaller than this threshold. We set the value to 0, and the gray

value of the other group is greater than this threshold. We set the value to 255 to obtain a binarized image.

E. Mask

Finally, to obscure unwanted edge information, we multiplied the image with a mask, as shown in Figure S5. Through the above steps, we can automatically capture information on retinal blood vessels.

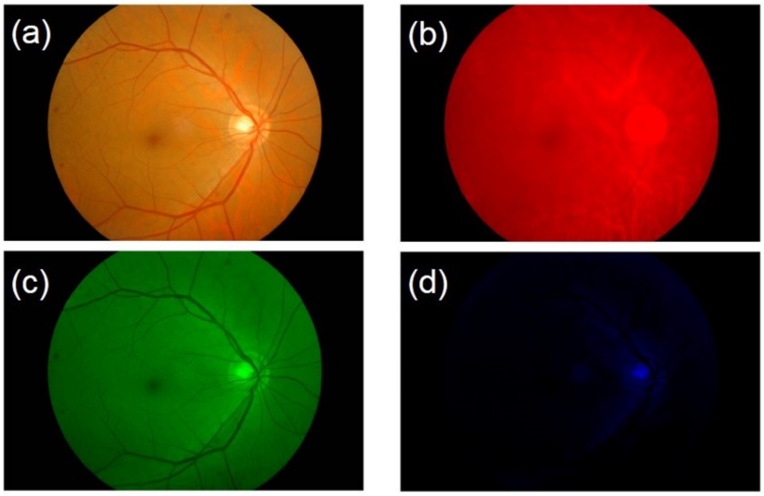

Figure S3. (**a**) Color retinal image; (**b**) R channel retinal image; (**c**) G channel retinal image; (**d**) B channel retinal image.

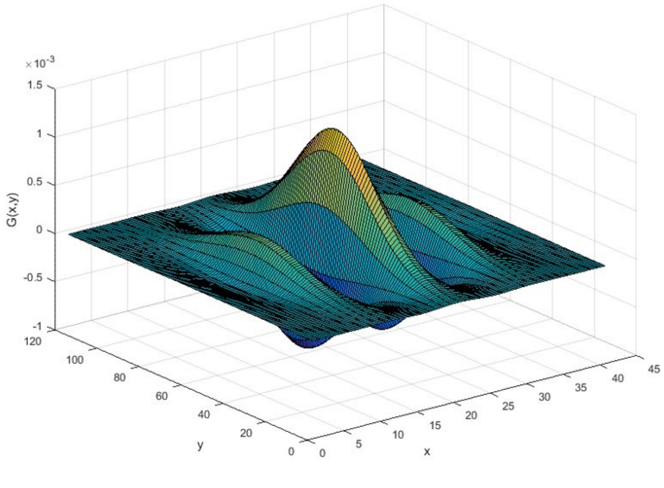

Figure S4. Two-dimensional Gabor filter schematic.

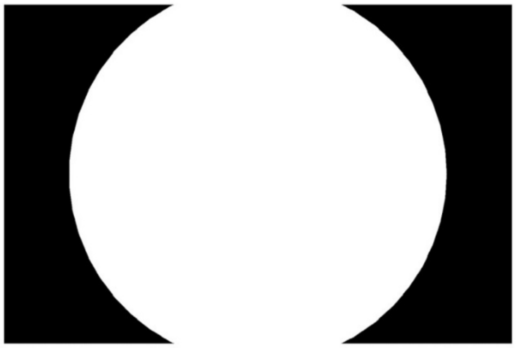

Figure S5. Mask diagram.

S5. Definition of Sensitivity, Precision, F1-Score, and Accuracy

Table S1 is a confusion matrix of binary classification.

Actual is the answer to your information, and *Predict* is the result predicted by the model. Moreover, *Positive* is "true" and is usually used in medicine as "onset." *Negative* is a "fake." In medicine, "no disease" is usually used. True positive: The real situation is "yes," and the model says "yes." True negative (TN): The real situation is "yo," and the model says "no." False positive (FP): The real situation is "no," and the model says "yes." False negative (FN): The real situation is "yes," and the model says "no." The Formula (S1) is sensitivity. Sensitivity is equal to recall, which is "the disease detection rate," so a high value is preferred. The Formula (S2) is precision, which is the ratio of the model diagnosis that is sick and has a disease. A high value is preferred. The Formula (S3) is accuracy, which is the correct rate of the model's overall judgment. A high value is preferred. Formula (S4) F1-score: If today I think both precision and recall are equally important, I want to use an indicator to integrate it. This value is the F1-score, which is the result of the harmonic averaging method.

Table S1. Confusion matrix of binary classification.

		**Predict**		
		**T**	**F**	
Actual	T	True Positive (TP)	False Negative (FN)	Actual Positive (TP + FN)
	F	False Positive (FP)	True Negative (TN)	Actual Negative (FP + TN)
		Predicted Positive (TP + FP)	Predicted Negative (FN + TN)	TP + FP + FN + TN

$$Sensitivity = \frac{\mathrm{TP}}{\mathrm{TP+FN}}. \quad \text{(S8)}$$

$$\mathrm{Precision} = \frac{\mathrm{TP}}{\mathrm{TP+FP}}. \quad \text{(S9)}$$

$$Accuracy = \frac{\mathrm{TP+TN}}{\mathrm{TP+FP+FN+TN}}. \quad \text{(S10)}$$

$$F1-score = \frac{2 \times \mathrm{Sensitivity} \times \mathrm{Precision}}{\mathrm{Sensitivity} + \mathrm{Precision}}. \quad \text{(S11)}$$

Reference

[R1] Yannuzzi, L. A. *et al.* Fluorescein Angiography Complication Survey. *Ophthalmology* **93**(5), 611-617(1986).

[R2] Littmann H. Die Zeiss Funduskamera Ber. 59. Zusammenkunft Deutsch. Ophthalmolog. Gesellsch., Heidelberg 1955 (Verlag Bergmann, Munchen). p. 318.

[R3] Huang, D. *et al.* Optical coherence tomography. *Science* **254**, 1178-1181 (1991).

[R4] Morgner, U. *et al.* Spectroscopic optical coherence tomography. *Optics letters* **25**(2) 111-113(2000).

[R5] Fercher, A. F., Hitzenberger, C. K., Drexler, W., Kamp, G. & Sattmann, H. In vivo optical coherence tomography. *Am J Ophthalmol* **116**, 113–114(1993).

[R6] Izatt, J. A. *et al.* Micrometer-scale resolution imaging of the anterior eye in vivo with optical coherence tomography. *Archives of ophthalmology* **12**(2), 1584-1589(1994).

[R7] Bagayev, S. N. *et al.* Optical coherence tomography for in situ monitoring of laser corneal ablation. *Journal of biomedical optics* **7**(4), 633-642(2002).

© 2020 by the authors. Submitted for possible open access publication under the terms and conditions of the Creative Commons Attribution (CC BY) license (http://creativecommons.org/licenses/by/4.0/).
